# Canagliflozin and iron metabolism in the CREDENCE trial

**DOI:** 10.1093/ndt/gfae198

**Published:** 2024-09-20

**Authors:** Akihiko Koshino, Hiddo J L Heerspink, Niels Jongs, Sunil V Badve, Clare Arnott, Bruce Neal, Meg Jardine, Kenneth W Mahaffey, Carol Pollock, Vlado Perkovic, Michael K Hansen, Stephan J L Bakker, Takashi Wada, Brendon L Neuen

**Affiliations:** Department of Clinical Pharmacy and Pharmacology, University Medical Center Groningen, University of Groningen, Groningen, The Netherlands; Department of Nephrology and Rheumatology, Kanazawa University, Ishikawa, Japan; Department of Clinical Pharmacy and Pharmacology, University Medical Center Groningen, University of Groningen, Groningen, The Netherlands; The George Institute for Global Health, UNSW Sydney, Sydney, Australia; Department of Clinical Pharmacy and Pharmacology, University Medical Center Groningen, University of Groningen, Groningen, The Netherlands; The George Institute for Global Health, UNSW Sydney, Sydney, Australia; Department of Nephrology, St George Hospital, Sydney, Australia; Faculty of Medicine, University of New South Wales, Sydney, Australia; The George Institute for Global Health, UNSW Sydney, Sydney, Australia; Department of Cardiology, Royal Prince Alfred Hospital, Sydney, Australia; The George Institute for Global Health, UNSW Sydney, Sydney, Australia; School of Public Health, Imperial College London, UK; The George Institute for Global Health, UNSW Sydney, Sydney, Australia; NHMRC Clinical Trials Centre University of Sydney NSW, Sydney, Australia; Concord Repatriation General Hospital, Sydney, Australia; Stanford Center for Clinical Research, Stanford University School of Medicine, Stanford, CA, USA; Kolling Institute of Medical Research, Sydney Medical School, University of Sydney, Sydney, Australia; Royal North Shore Hospital, St Leonards, New South Wales, Australia; The George Institute for Global Health, UNSW Sydney, Sydney, Australia; Faculty of Medicine, University of New South Wales, Sydney, Australia; Janssen Research & Development, LLC, Spring House, PA, USA; Department of Internal Medicine, University Medical Center Groningen, University of Groningen, Groningen, The Netherlands; Department of Nephrology and Rheumatology, Kanazawa University, Ishikawa, Japan; The George Institute for Global Health, UNSW Sydney, Sydney, Australia; Royal North Shore Hospital, St Leonards, New South Wales, Australia

**Keywords:** anemia, cardiovascular, chronic kidney disease, iron deficiency, sodium-glucose cotransporter 2 inhibitors

## Abstract

**Background:**

Studies in patients with heart failure have indicated that sodium-glucose cotransporter 2 (SGLT2) inhibitors increase iron use and enhance erythropoiesis. In this *post hoc* analysis of the Canagliflozin and Renal Endpoints in Diabetes with Established Nephropathy Clinical Evaluation (CREDENCE) trial, we evaluated the effects of canagliflozin on iron metabolism in patients with chronic kidney disease (CKD) and whether the effects of canagliflozin on hemoglobin and cardiorenal outcomes were modified by iron deficiency.

**Methods:**

We measured serum iron, total iron binding capacity (TIBC), transferrin saturation (TSAT) and ferritin at baseline and 12 months. The effects of canagliflozin, relative to placebo, on iron markers were assessed with analysis of covariance. Interactions between baseline iron deficiency, defined as TSAT <20%, and the effects of canagliflozin on hemoglobin and cardiorenal outcomes were evaluated with mixed effect models and Cox regression models, respectively.

**Results:**

Of 4401 participants randomized in CREDENCE, 2416 (54.9%) had iron markers measured at baseline, of whom 924 (38.2%) were iron deficient. Canagliflozin, compared with placebo, increased TIBC by 2.1% [95% confidence interval (CI) 0.4, 3.8; *P* = .014] and decreased ferritin by 11.5% (95% CI 7.1, 15.7; *P* < .001) with no clear effect on serum iron or TSAT. Canagliflozin increased hemoglobin over the trial duration by 7.3 g/L (95% CI 6.2, 8.5; *P* < .001) and 6.7 g/L (95% CI 5.2, 8.2; *P* < .001) in patients with and without iron deficiency, respectively (*P* for interaction = .38). The relative effect of canagliflozin on the primary outcome of doubling of serum creatinine, kidney failure or death due to cardiovascular disease or kidney failure (hazard ratio 0.70, 95% CI 0.56, 0.87) was consistent regardless of iron deficiency (*P* for interaction = .83), as were effects on other cardiovascular and mortality outcomes (all *P* for interactions ≥0.10).

**Conclusion:**

Iron deficiency is highly prevalent in patients with type 2 diabetes and CKD. Canagliflozin increased TIBC and decreased ferritin in patients with type 2 diabetes and CKD, suggesting increased iron utilization, and improved hemoglobin levels and clinical outcomes regardless of iron deficiency.

## Graphical Abstract

**Graphical Abstract:**
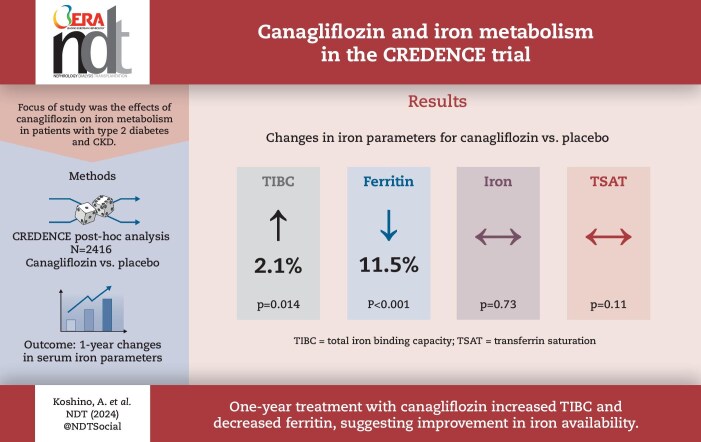

KEY LEARNING POINTS **What was known:**

- Sodium-glucose cotransporter 2 (SGLT2) inhibitors reduce the risk of cardiovascular events and chronic kidney disease (CKD) progression in patients with CKD.
- In patients with heart failure, the SGLT2 inhibitor dapagliflozin increased red blood cell mass and improved iron availability.
- In this *post hoc* analysis of the CREDENCE trial, we assessed the effect of canagliflozin on circulating iron markers in patients with CKD and type 2 diabetes.

**This study adds:**

- This study found that canagliflozin reduced ferritin and increased total iron binding capacity over 12 months compared with placebo.
- Canagliflozin increased hemoglobin and corrected anemia regardless of iron deficiency, defined as transferrin saturation <20% at baseline.
- The risk reductions for cardiovascular, kidney and mortality outcomes with canagliflozin were also consistent across baseline iron deficiency.

**Potential impact:**

- These findings suggest that canagliflozin increased iron utilization in patients with CKD and type 2 diabetes.
- Our results support future studies to assess the interaction between SGLT2 inhibitors, ferrokinetics, and iron therapies in this population.

## INTRODUCTION

Disordered iron metabolism and anemia occur commonly in patients with type 2 diabetes and chronic kidney disease (CKD) [1–3]. Large observational studies in patients with CKD have indicated that iron deficiency is independently associated with cardiovascular events and all-cause death, regardless of anemia [3, 4]. Large-scale randomized trials in patients with heart failure have shown that intravenous iron administration reduces the risk of hospitalization for heart failure or cardiovascular death, independent of hemoglobin levels [5]. In patients with dialysis-dependent kidney failure, a proactive high-dose intravenous iron treatment regimen showed a lower risk of major cardiovascular events or death as compared with a reactive low-dose regimen [6]. These data suggest iron deficiency, whether absolute or functional, may have an important impact on cardiovascular outcomes in patients with CKD or heart failure.

In addition to their benefits on renal and cardiovascular outcomes including heart failure, *post hoc* analyses of large clinical trials demonstrated that sodium-glucose cotransporter 2 (SGLT2) inhibitors have favorable effect on anemia [7–9]. Previously these changes in hematological parameters were thought to reflect hemoconcentration. However, a recent mechanistic study revealed that the increase in urine output by SGLT2 inhibitors is only acute and transient [10]. In contrast to this, the increase in hemoglobin is gradual and peaks around 4 months [8, 9]. In addition, another clinical study observed a rise in reticulocyte count with dapagliflozin [11]. These data suggest that erythropoiesis induced by SGLT2 inhibitors at least partly contributes to the increase in red blood cell mass.

In patients with heart failure with reduced ejection fraction, dapagliflozin decreased circulating transferrin saturation (TSAT), ferritin and hepcidin, and increased total iron binding capacity (TIBC), suggesting that SGLT2 inhibition might increase iron use by addressing hepcidin-induced functional iron blockade [12]. In a mechanistic randomized trial in patients with heart failure, empagliflozin reduced ferritin and increased myocardial iron content on cardiac magnetic resonance imaging [13]. Together with evidence demonstrating the benefits of iron administration in heart failure, these effects of SGLT2 inhibitors on markers of iron metabolism suggest that improvements in iron utilization may contribute to their clinical benefits on heart failure–related outcomes [14]. However, there are few, if any, data regarding the impact of SGLT2 inhibitors on iron metabolism in patients with CKD. Any effects of SGLT2 inhibitors on iron metabolism in CKD would be important to understand, given the high prevalence of iron deficiency and anemia in this population, especially as kidney function declines [3, 15].

In this *post hoc* analysis of the Canagliflozin and Renal Endpoints in Diabetes with Established Nephropathy Clinical Evaluation (CREDENCE) trial, we evaluated the effect of 1-year treatment with the SGLT2 inhibitor canagliflozin on serum iron, TIBC, TSAT and ferritin levels in patients with type 2 diabetes and CKD. We also assessed whether the effects of canagliflozin on hemoglobin levels and cardiorenal outcomes were modified by the presence of iron deficiency at baseline.

## MATERIALS AND METHODS

### Participants and study procedure

CREDENCE was a multinational, randomized, double-blinded, placebo-controlled, event-driven trial that enrolled patients from 690 sites in 34 countries from March 2014 to 2018. The study protocol and main results of the trial have been published previously [16, 17]. Briefly, individuals with diabetes and CKD were randomized (1:1) to receive canagliflozin 100 mg/day or a matching placebo. Key inclusion criteria were: age ≥30 years, type 2 diabetes, estimated glomerular filtration rate (eGFR) 30 to <90 mL/min/1.73 m^2^, urinary albumin-to-creatinine ratio (UACR) >300–5000 mg/g and glycated hemoglobin (HbA1c) ≥6.5%–12.0%. All patients were required to be receiving stable maximum-labeled or tolerated dose of renin–angiotensin system blockade for at least 4 weeks prior to randomization. Patients with suspected non-diabetic kidney disease or type 1 diabetes were excluded. CREDENCE was conducted according to the principles of the Declaration of Helsinki and was registered with ClinicalTrials.gov (NCT02065791). Ethics committees at all participating sites approved the protocol and all participants provided written informed consent. Subjects who agreed to participate in exploratory biomarker evaluation, including the current study, gave separate written informed consent.

### Measurement of iron parameters and definition of iron deficiency

Serum samples of CREDENCE participants who agree to the exploratory biomarker evaluations were collected at baseline and Week 52 after randomization and were immediately stored at –80°C until measurement. We measured serum iron, TIBC, TSAT and ferritin levels in these samples. The measurements were performed with established clinical laboratory assays (Cobas IRON2 and Cobas Elecsys Ferritin; Roche Diagnostics, Mannheim, Germany) at University Medical Center Groningen between December 2022 and March 2023.

Iron deficiency was defined as TSAT <20% based on the Kidney Disease: Improving Global Outcomes (KDIGO) practice guideline for anemia in CKD and other literature [2, 18, 19]. In addition, we subdivided iron deficiency into “absolute iron deficiency” (TSAT <20% and ferritin <100 μg/L) and “functional iron deficiency” (TSAT <20% and ferritin ≥100 μg/L) [2, 18].

### Outcomes

#### Primary and secondary trial outcomes

The primary outcome of the CREDENCE trial was a composite of kidney failure (sustained eGFR <15 mL/min/1.73 m^2^, maintenance dialysis or kidney transplantation), doubling of the serum creatinine, or death from cardiovascular disease or kidney failure. Secondary outcomes included: cardiovascular death or hospitalization for heart failure; major adverse cardiovascular events (cardiovascular death, nonfatal myocardial infarction or nonfatal stroke); a kidney-specific composite (primary outcome excluding cardiovascular death); and all-cause mortality. All outcomes were adjudicated by independent blinded committees.

#### Anemia-related outcomes

Hemoglobin concentrations and hematocrit levels were measured at baseline and every 52 weeks after randomization. All measurements were carried out by a central laboratory. Anemia was defined by using hemoglobin thresholds (<130 g/L in men or <120 g/L in women) according to the KDIGO and WHO guidelines [18, 20]. We defined anemia correction as a hemoglobin concentration above these thresholds at a follow-up visit among patients with anemia at baseline. Conversely, anemia onset was defined as a hemoglobin measurement below these thresholds in participants without baseline anemia.

### Statistical analysis

All analyses presented here followed the intention-to-treat principle. Baseline characteristics by treatment allocation and baseline iron deficiency are presented according to their distribution as mean (standard deviation), median (25%, 75% percentile point) or *n* (%).

We used analysis of covariance with adjustment for log-transformed baseline level to assess the placebo-corrected effect of canagliflozin on relative changes in iron markers from baseline to Week 52. We also determined the effect of canagliflozin on iron parameters across key subgroups, including baseline iron deficiency (TSAT <20% or ≥20%), sex, anemia (yes or no), eGFR (≤60 or >60 mL/min/1.73 m^2^) and UACR (≤1000 or >1000 mg/g). We calculated Pearson's correlation coefficients to determine the relationship between changes in iron parameters and hemoglobin and hematocrit from baseline to Week 52.

The effects of canagliflozin on change in hemoglobin and hematocrit from baseline were assessed using a mixed effects model for repeated measures with a restricted maximum likelihood estimator. The model consisted of the fixed categorical effects of randomized treatment, trial visit, eGFR category at screening and the interaction of treatment-by-visit, and the fixed continuous covariates of baseline value and the baseline-by-visit interaction. An unstructured covariance matrix was applied to estimate within-patient errors. Potential effect modification by iron deficiency at baseline was tested by adding the main effect for iron deficiency subgroup (yes or no) and all two-way and three-way interaction terms between treatment, iron deficiency and trial visit to the relevant models. We also described the proportion of patients with absolute or functional iron deficiency at baseline and Week 52, with the proportion of patients with iron deficiency at Week 52 by treatment allocation compared using chi-square tests.

The effects of canagliflozin on anemia correction (among patients with anemia at baseline) and anemia onset (among patients without anemia at baseline) were assessed using Cox proportional-hazards regression models. The analyses were stratified by screening eGFR categories consistent with the primary trial publication [16]. We investigated the consistency of effect of canagliflozin on anemia correction and anemia onset across baseline iron deficiency subgroups. The proportional hazards assumption was checked by inspection of Kaplan–Meier plots and by testing the independence between scaled Schoenfeld residuals and time.

Finally, we assessed the effects of canagliflozin on kidney and cardiovascular outcomes by iron deficiency at baseline. We used Cox regression models with the same stratification approach used in the analysis on anemia-related outcomes.

All analysis was performed with R version 4.2.1 (R Foundation for Statistical Computing, Vienna, Austria).

## RESULTS

### Baseline characteristics

Of 4401 CREDENCE participants, 2416 (54.9%) had available data on iron biomarkers at baseline (Supplementary data, Fig. S1). Mean age was 63.2 years; 811 (33.6%) were female; 302 (12.5%) had a history of heart failure; mean eGFR was 56.8 mL/min/1.73 m^2^; median UACR was 901 mg/g; and 924 (38.2%) had iron deficiency. Of those, 500 (54.1%) had absolute iron deficiency (TSAT <20% and ferritin <100 μg/L), 373 (40.4%) had functional iron deficiency (TSAT <20% and ferritin ≥100 μg/) and 51 (5.5%) had no ferritin measurement. Characteristics of participants according to iron deficiency at baseline are displayed in Table 1. Compared with patients without iron deficiency, those with iron deficiency were more likely to be female, and to have a history of heart failure, higher body mass index, and lower hemoglobin and hematocrit. The proportion of patients treated with iron products or erythropoiesis-stimulating agents at baseline was higher in iron-deficient patients, although the use of erythropoietin-stimulating agents was low overall (0.7%). Participant characteristics within iron deficiency subgroups were well balanced between canagliflozin and placebo.

**Table 1:** Baseline characteristics of CREDENCE participants according to the presence of iron deficiency and randomized treatment assignment.

	No iron deficiency			Iron deficiency		
	Total	Canagliflozin	Placebo	Total	Canagliflozin	Placebo
	*N* = 1492	*N* = 745	*N* = 747	*N* = 924	*N* = 472	*N* = 452
Age, years	63.3 (9.1)	63.1 (9.1)	63.5 (9.1)	63.1 (9.3)	63.3 (9.2)	62.9 (9.4)
Women, *n* (%)	384 (25.7)	193 (25.9)	191 (25.6)	427 (46.2)	222 (47.0)	205 (45.4)
Race, *n* (%)						
White	1064 (71.3)	531 (71.3)	533 (71.4)	670 (72.5)	354 (75.0)	316 (69.9)
Black	69 (4.6)	37 (5.0)	32 (4.3)	65 (7.0)	32 (6.8)	33 (7.3)
Asian	217 (14.5)	106 (14.2)	111 (14.9)	84 (9.1)	37 (7.8)	47 (10.4)
Other	142 (9.5)	71 (9.5)	71 (9.5)	105 (11.4)	49 (10.4)	56 (12.4)
Current smoker, *n* (%)	222 (14.9)	115 (15.4)	107 (14.3)	129 (14.0)	70 (14.8)	59 (13.1)
CV disease, *n* (%)	759 (50.9)	378 (50.7)	381 (51.0)	478 (51.7)	247 (52.3)	231 (51.1)
Heart failure, *n* (%)	169 (11.3)	85 (11.4)	84 (11.2)	133 (14.4)	59 (12.5)	74 (16.4)
Diabetes duration, years	15.9 (8.8)	15.7 (8.9)	16.1 (8.7)	16.4 (8.5)	16.3 (8.6)	16.5 (8.5)
BMI, kg/m^2^	31.2 (5.9)	31.1 (5.9)	31.3 (5.9)	32.7 (6.7)	32.8 (6.9)	32.6 (6.6)
sBP, mmHg	140.3 (15.6)	140.0 (15.7)	140.5 (15.5)	140.4 (16.2)	140.9 (16.5)	139.8 (15.9)
Hemoglobin, g/L	135.4 (16.8)	136.1 (17.1)	134.7 (16.5)	127.8 (16.2)	128.2 (16.6)	127.3 (15.7)
Anemia, *n* (%)	403 (27.0)	184 (24.7)	219 (29.3)	385 (41.7)	196 (41.5)	189 (41.8)
Hematocrit, %	41.2 (5.2)	41.4 (5.3)	41.0 (5.1)	39.3 (5.0)	39.4 (5.2)	39.1 (4.8)
HbA1c, %	8.2 (1.3)	8.2 (1.3)	8.2 (1.3)	8.3 (1.3)	8.3 (1.3)	8.3 (1.3)
LDL cholesterol, mg/dL	96.8 (41.0)	97.3 (43.2)	96.4 (38.8)	92.5 (41.3)	92.4 (42.1)	92.6 (40.5)
eGFR, mL/min/1.73 m^2^	56.9 (18.2)	56.9 (18.2)	56.8 (18.3)	56.8 (18.2)	56.7 (18.1)	56.8 (18.3)
Screening eGFR, *n* (%)						
30–<45	428 (28.7)	219 (29.4)	209 (28.0)	251 (27.2)	134 (28.4)	117 (25.9)
45–<60	445 (29.8)	214 (28.7)	231 (30.9)	288 (31.2)	148 (31.4)	140 (31.0)
60–<90	619 (41.5)	312 (41.9)	307 (41.1)	385 (41.7)	190 (40.3)	195 (43.1)
Median UACR, mg/g	928 (472–1783)	932 (487–1792)	927 (461–1764)	864 (453–1665)	882 (443–1682)	832 (471–1653)
Median TSAT, %	26 (23–31)	26 (23–31)	25 (23–31)	15 (12–17)	15 (12–18)	15 (13–17)
Median ferritin, µg/L^a^	177 (100–311)	182 (103–319)	176 (97–304)	86 (43–164)	87 (42–165)	86 (44–159)
Anemia medication, *n* (%)						
Iron preparation	62 (4.2)	35 (4.7)	27 (3.6)	54 (5.8)	26 (5.5)	28 (6.2)
ESAs	8 (0.5)	4 (0.5)	4 (0.5)	8 (0.9)	4 (0.8)	4 (0.9)

Data are presented as mean (standard deviation), median (interquartile range) or *n* (%).

^a^122 subjects (51 without iron deficiency and 71 with iron deficiency) had no ferritin measurement due to insufficient serum samples.

Iron deficiency was defined as TSAT <20% according to the KDIGO guideline. Anemia was defined as hemoglobin <130 g/L in men or <120 g/L in women.

CV, cardiovascular; BMI, body mass index; ESAs, erythropoiesis-stimulating agents; LDL, low-density lipoprotein; sBP, systolic blood pressure.

### Effect of canagliflozin on iron markers

**Figure 1:**
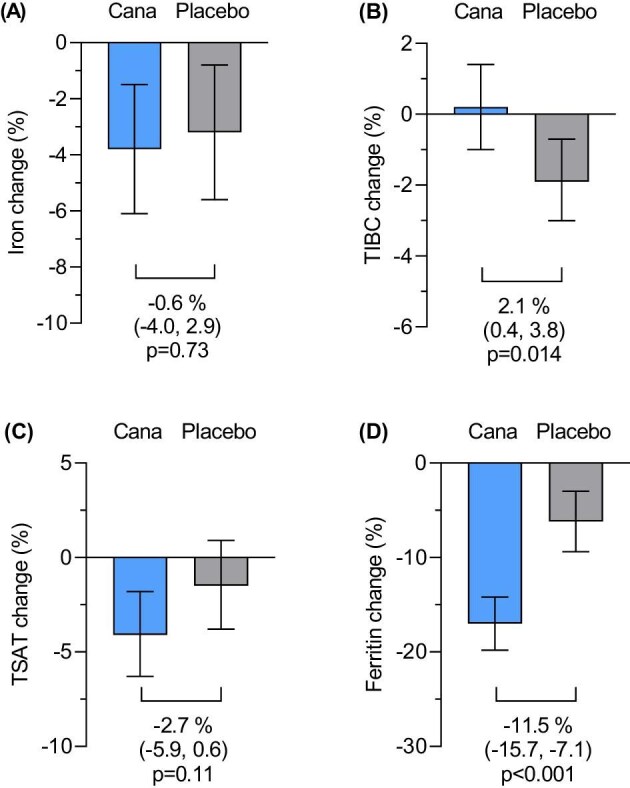
Percent changes in (**A**) iron, (**B**) TIBC, (**C**) TSAT and (**D**) Ferritin in the canagliflozin and placebo group from baseline to Week 52. Effects of canagliflozin on changes in iron biomarkers were estimated with analysis of covariance adjusted for log-transformed value at baseline. Cana, canagliflozin.

Percentage changes in iron parameters by randomized treatment are displayed in Fig. 1. Relative to placebo, canagliflozin significantly increased TIBC by 2.1% [95% confidence interval (CI) 0.4, 3.8; *P* = .014] and decreased ferritin by 11.5% (95% CI 7.1, 15.7; *P* < .001). There were no significant effects on iron concentration (–0.6%, 95% CI –4.0, 2.9; *P* = .73) and TSAT levels (–2.7%, 95% CI –5.9, 0.6; *P* = .11).

The effects of canagliflozin on iron markers were generally consistent across iron deficiency (Table 2), sex, anemia, eGFR and UACR subgroups (Supplementary data, Table S1). There was some evidence that the effect of canagliflozin on TIBC was more pronounced in patients with iron deficiency (*P* for interaction = 0.04). The proportion of patients with iron deficiency at Week 52 was similar between canagliflozin and placebo groups (41.9% vs 38.5%, *P* = .16; Supplementary data, Fig. S2). At Week 52, among participants randomized to canagliflozin, the decrease in ferritin and the increase in TIBC were correlated with increases in hemoglobin and hematocrit (all *P* < .05; Supplementary data, Table S2).

**Table 2:** Percent changes in iron markers in the canagliflozin and placebo group from baseline to Week 52 by iron deficiency at baseline.

	Geometric mean at baseline (95% CI)		Geometric mean at Week 52 (95% CI)		Change from baseline to Week 52, % (95% CI)				
Biomarker	Cana	Placebo	Cana	Placebo	Cana	Placebo	Effect, % (95% CI)^a^	*P*	*P* for int
Iron (µmol/L)									
Overall (*N* = 2013)	13.1 (12.7, 13.4)	13.1 (12.8, 13.4)	12.6 (12.2, 12.9)	12.7 (12.4, 13.0)	–3.8 (–6.1, –1.5)	–3.2 (–5.6, –0.8)	–0.6 (–4.0, 2.9)	.73	
Iron deficient									
No (*n* = 1236)	16.2 (15.9, 16.6)	15.8 (15.4, 16.1)	14.0 (13.6, 14.5)	13.8 (13.5, 14.3)	–13.1 (–15.6, –10.5)	–13.0 (–15.5, –10.4)	–0.1 (–4.1, 4.1)	.97	.64
Yes (*n* = 777)	9.3 (9.0, 9.7)	9.7 (9.3, 10.0)	10.6 (10.2, 11.1)	10.9 (10.5, 11.4)	12.8 (8.2, 17.6)	14.8 (10.0, 19.9)	–1.8 (–7.5, 4.3)	.56	
TIBC (µmol/L)									
Overall (*N* = 2004)	62.5 (61.7, 63.3)	62.2 (61.3, 63.1)	62.5 (61.6, 63.4)	61.1 (60.3, 61.9)	0.2 (–1.0, 1.4)	–1.9 (–3.0, –0.7)	2.1 (0.4, 3.8)	.014	
Iron deficient									
No (*n* = 1232)	59.8 (58.9, 60.7)	58.8 (57.8, 59.9)	59.8 (58.8, 60.8)	58.8 (57.9, 59.8)	0.3 (–1.1, 1.7)	–0.4 (–1.7, 1.0)	0.7 (–1.3, 2.7)	.49	.04
Yes (*n* = 772)	66.8 (65.4, 68.2)	68.2 (66.8, 69.5)	67.0 (65.4, 68.7)	65.0 (63.6, 66.4)	–0.1 (–2.1, 2.0)	–4.2 (–6.2, –2.1)	4.3 (1.3, 7.4)	.005	
TSAT (%)									
Overall (*N* = 2003)	20.9 (20.4, 21.5)	21.1 (20.6, 21.6)	20.1 (19.5, 20.7)	20.7 (20.2, 21.3)	–4.1 (–6.3, –1.8)	–1.5 (–3.8, 0.9)	–2.7 (–5.9, 0.6)	.11	
Iron deficient									
No (*n* = 1231)	27.2 (26.7, 27.6)	26.9 (26.4, 27.4)	23.4 (22.7, 24.1)	23.5 (22.9, 24.2)	–13.6 (–15.9, –11.1)	–12.6 (–15.0, –10.2)	–1.1 (–4.9, 2.9)	.59	.24
Yes (*n* = 772)	14.0 (13.5, 14.4)	14.1 (13.7, 14.6)	15.9 (15.1, 16.7)	16.8 (16.1, 17.6)	13.3 (8.6, 18.1)	19.4 (14.3, 24.7)	–5.1 (–10.7, 0.8)	.09	
Ferritin (µg/L)									
Overall (*N* = 1834)	130.5 (122.7, 138.7)	129.4 (121.5, 137.7)	108.2 (101.7, 115.1)	121.4 (114.2, 129.0)	–17.0 (–19.8, –14.2)	–6.2 (–9.4, –3.0)	–11.5 (–15.7, –7.1)	<.001	
Iron deficient									
No (*n* = 1139)	175.0 (163.5, 187.4)	167.5 (156.1, 179.7)	142.6 (132.8, 153.2)	151.2 (141.1, 162.1)	–18.2 (–21.3, –15.0)	–10.0 (–13.5, –6.4)	–9.1 (–14.0, –4.0)	.001	.15
Yes (*n* = 695)	81.2 (73.5, 89.6)	84.2 (75.9, 93.5)	69.2 (62.9, 76.2)	84.3 (76.0, 93.4)	–15.1 (–20.2, –9.7)	0.5 (–5.7, 7.1)	–15.5 (–22.7, –7.7)	<.001	

^a^Placebo-corrected effects of canagliflozin on iron biomarkers were estimated with analysis of covariance adjusted for log-transformed value at baseline.

Iron deficiency was defined as TSAT <20%.

Cana, canagliflozin.

### Effect of canagliflozin on hemoglobin levels and anemia by iron deficiency

**Figure 2:**
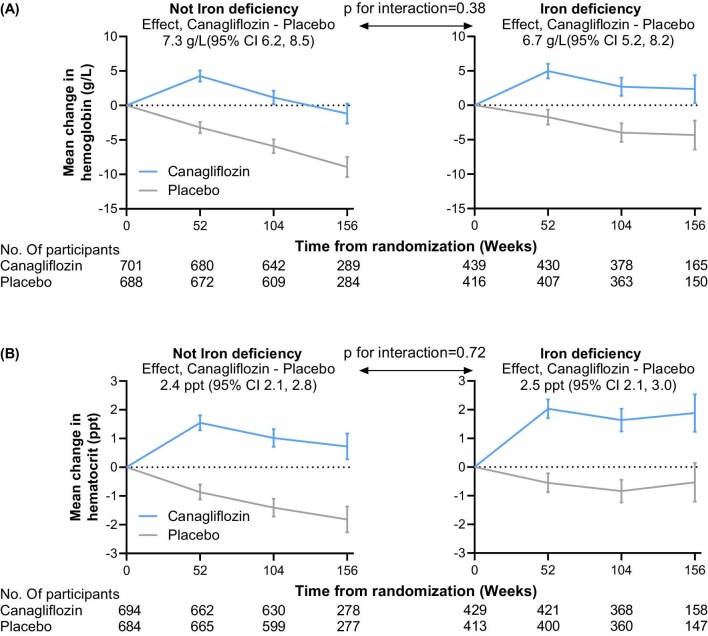
Effects of canagliflozin on (**A**) hemoglobin and (**B**) hematocrit over time by iron deficiency at baseline. Linear mixed-effects models with a restricted maximum likelihood estimator calculated the least-squares mean changes from baseline in hemoglobin and hematocrit. The model included fixed, categorical effects of therapy, trial visit, eGFR at screening and treatment-by-visit interaction along with fixed, continuous baseline value variables and baseline value by visit interaction. Effect modification by iron deficiency at baseline was tested by adding the main effect for iron deficiency subgroup (yes or no) and all two-way and three-way interaction terms between treatment, iron deficiency and trial visit to the relevant models. An unstructured covariance structure was used to model the within-patient errors. Ppt, percentage points.

The effects of canagliflozin on hemoglobin and hematocrit by baseline iron deficiency are displayed in Fig. 2. In the placebo group, hemoglobin decreased during follow-up, with mean changes from baseline of –5.1 g/L (95% CI –5.9, –4.3; *P* < .001) and –2.9 g/L (95% CI –4.0, –1.9; *P* < .001) in the non-iron deficient and iron deficient patients, respectively. Canagliflozin significantly increased hemoglobin, relative to placebo, regardless of iron deficiency at baseline. The mean between treatment group difference over time was 7.3 g/L (95% CI 6.2, 8.5; *P* < .001) in patients without iron deficiency and 6.7 g/L (95% CI 5.2, 8.2; *P* < .001) in patients with iron deficiency (*P* for interaction = 0.38; Fig. 2A). Similarly, canagliflozin increased hematocrit regardless of iron deficiency [mean absolute difference 2.4% (95% CI 2.1, 2.8), *P* < .001 in the non-iron deficient group; 2.5% (95% CI 2.1, 3.0), *P* < .001 in the iron deficient group; *P* for interaction = 0.72; Fig. 2B]. Similar results were observed when analyses were restricted to patients with eGFR ≤60 mL/min/1.73 m^2^ (Supplementary data, Table S3).

The effects of canagliflozin on correction of anemia and incident anemia are shown in Supplementary data, Table S4. Among 771 participants with anemia at baseline, canagliflozin increased the likelihood of anemia correction [316.6 vs 132.1 per 1000 person-years; hazard ratio (HR) 2.55 (95% CI 2.01, 3.24), *P* < .001]. This effect was consistent irrespective of baseline iron deficiency (*P* for interaction = 0.78). Conversely, among 1416 patients without baseline anemia, canagliflozin reduced the risk of incident anemia compared with placebo [97.7 vs 178.4 per 1000 person-years; HR 0.51 (95% CI 0.41, 0.62), *P* < .001], with effects consistent across groups with and without iron deficiency (*P* for interaction = .31).

### Effect of canagliflozin on cardiovascular and kidney outcomes by iron deficiency

**Figure 3:**
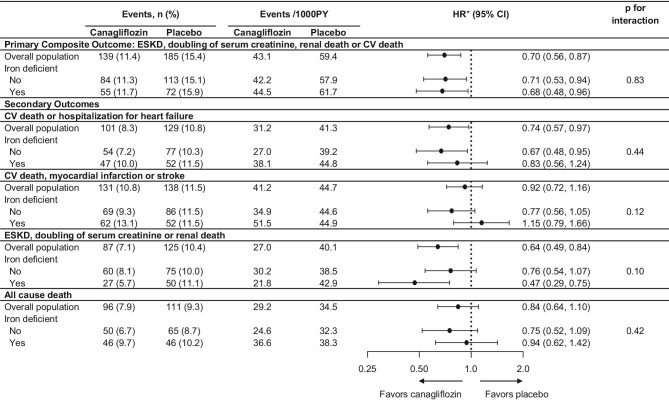
Effects of canagliflozin on cardiovascular, kidney and mortality outcomes by iron deficiency at baseline. *HR of canagliflozin group, comparing with placebo group, on trial outcomes. Iron deficiency was defined as transferrin saturation <20%. The Cox proportional hazard regression model was stratified with prespecified eGFR strata at screening. At baseline, 924 were iron deficient (472 in the canagliflozin group and 452 in the placebo group) and 1492 were not iron deficient (745 in the canagliflozin group and 747 in the placebo group). ESKD, end- stage kidney disease; CV, cardiovascular.

Figure 3 summarizes the effect of canagliflozin on clinical outcomes overall and by baseline iron deficiency. In the placebo arm, event rates for cardiovascular, kidney and mortality outcomes were higher in patients with iron deficiency compared with those without iron deficiency. During a median follow-up of 2.8 years, canagliflozin reduced the risk of the primary cardiorenal composite outcome by 30% [43.1 vs 59.4 per 1000 person-years; HR 0.70 (95% CI 0.56, 0.87), *P* = .002]. This effect was consistent across patients with and without iron deficiency at baseline (*P* for interaction = .83). There was no significant interaction between treatment and baseline iron deficiency on the secondary cardiovascular, kidney or mortality outcomes (all *P* for interactions ≥0.10).

## DISCUSSION

The effects of SGLT2 inhibitors on iron metabolism and erythropoiesis were not anticipated when these medicines were originally developed; however, it is increasingly recognized that SGLT2 inhibitors have many effects beyond improving glycemic control including potentially important effects on hematopoiesis. In this *post hoc* analysis of the CREDENCE trial, we observed that treatment with canagliflozin over 1-year increased TIBC and reduced ferritin levels, suggesting that canagliflozin may have increased iron utilization. Increases in hemoglobin and hematocrit with canagliflozin were similar regardless of the presence or absence of iron deficiency at baseline, as were the effects of canagliflozin on kidney, cardiovascular and mortality outcomes. These findings identify a clear role of SGLT2 inhibitors in iron metabolism, and further confirm the consistent clinical benefits of SGLT2 inhibitors across diverse patient subsets with type 2 diabetes and CKD.

Accumulating data indicate that SGLT2 inhibitors increase red blood cell mass, possibly by increasing iron utilization as well as increasing erythropoietin synthesis. Changes in TIBC and ferritin, which occurred in parallel with reductions in hepcidin and other markers of inflammation, suggest that SGLT2 inhibitors may promote iron mobilization through reducing systemic inflammation [14, 21]. This phenomenon has been observed previously in patients with heart failure with reduced ejection fraction and those with type 2 diabetes without CKD [12, 22]. In patients with type 2 diabetes and CKD, a *post hoc* analysis of the DELIGHT trial reported that changes in TSAT and transferrin over 24 weeks of treatment with dapagliflozin correlated with changes in urinary and plasma interleukin-6, an inflammatory cytokine that regulates hepcidin expression [23, 24]. These favorable effects on iron storage and mobilization may contribute to reductions in anemia-related outcomes reported with SGLT2 inhibitors in patients with CKD [7, 8].

Our results complement data in patients with heart failure with reduced ejection fraction, where dapagliflozin increased hemoglobin and hematocrit, and improved other anemia-related outcomes regardless of iron deficiency [12]. This effect on anemia is distinct from erythropoiesis-stimulating agents, which are less effective in patients with iron deficiency, and underscore the concept that SGLT2 inhibitors improve iron availability but may not worsen iron deficiency [14]. The relatively small effect of canagliflozin on TIBC and the non-significant effect on TSAT in the current study are compatible with this notion. Our findings extend previous observations of the effects of SGLT2 inhibitors on iron metabolism to patients with CKD, in whom anemia and iron deficiency are highly prevalent, especially as kidney function declines [25].

Iron is a fundamental element important in cardiac myocyte and mitochondrial function [26, 27]. Iron deficiency, irrespective of anemia, has been consistently shown to be strongly linked to the risk of cardiovascular events in people with CKD and heart failure [3, 28]. Several lines of evidence suggest that improvements in iron utilization may contribute to the beneficial cardiovascular effects of SGLT2 inhibitors, particularly for preventing heart failure. In a randomized trial, treatment with empagliflozin increased myocardial iron uptake as measured by cardiac magnetic resonance imaging in patients with heart failure without diabetes [13]. These improvements in myocardial iron content were associated with favorable changes in left ventricular mass, volumes and function [13]. Additionally, mediation analyses indicate that changes in hemoglobin and hematocrit are the most important statistical mediators of the benefits of SGLT2 inhibitors on cardiovascular outcomes [29–31]. Further research is necessary to assess the mechanistic link between the effect of canagliflozin on iron metabolism and their cardiovascular benefit in patients with CKD.

In keeping with the finding that canagliflozin increased hemoglobin levels irrespective of baseline iron deficiency, the effect of canagliflozin on cardiorenal outcomes was consistent regardless of iron deficiency. These results support the initiation of SGLT2 inhibitors in patients regardless of iron deficiency.

Our findings also raise the question of whether the increasing use of SGLT2 inhibitors necessitates more widespread screening for—and correction of—iron deficiency in people with heart failure or CKD. Given the effect of SGLT2 inhibitors on iron parameters, our findings raise the possibility that testing iron stores in patients after initiation of SGLT2 inhibitors might be warranted to detect and correct deficiency. Although canagliflozin did not significantly increase the proportion of iron deficiency at Week 52 compared with placebo, the incidence of absolute iron deficiency was numerically higher in the canagliflozin group. Iron supplementation might be necessary for cases with worsening absolute iron deficiency. Current international guidelines for patients with CKD recommend testing and correcting iron deficiency in patients with anemia [2, 18]. In patients with heart failure, major guidelines also recommend using intravenous iron to improve functional status and reduce hospitalization for heart failure [32, 33]. The use of SGLT2 inhibitors and intravenous iron in both CKD and heart failure suggests a potential area of therapeutic synergy. Future studies are required to assess the interaction between intravenous iron administration and SGLT2 inhibitors [34]. Future studies may provide additional information on the safety profile of SGLT2 inhibitors in terms of erythrocytosis and thrombosis. However, cardiovascular and kidney benefits of SGLT2 inhibitor were consistent regardless of the anemia at baseline in the large clinical outcome trials [8, 9].

The current study has limitations. First, we were not able to measure hepcidin concentrations to evaluate the relationship between canagliflozin, inflammation, intestinal iron absorption and functional iron deficiency. Also, some other factors involved in inflammation and erythropoiesis, such as high-sensitivity C-reactive protein, folic acid and vitamin B12, were not available. Second, we only measured iron parameters at 52 weeks after the initiation of randomized treatment and did not have earlier measurements. The effects of SGLT2 inhibitors on iron markers are likely to be present earlier as observed in other studies [22, 23]. Third, the presently used iron parameters including TSAT and ferritin does not provide a complete assessment of body iron status [2, 35]. Fourth, the current analyses did not account for the initiation of iron supplementation or erythropoietin-stimulating agents after randomization. However, given the lower hemoglobin in the placebo group during follow-up, it may be that iron supplementation was initiated more frequently in the placebo group than in the canagliflozin group. Thus, the effect of canagliflozin on anemia in patients with iron deficiency may be underestimated. Finally, this was a *post hoc* study of about half of the patients in the CREDENCE trial and should be interpreted in such a light. However, the consistency of our results with previous studies on the effects of SGLT2 inhibitors on iron metabolism in heart failure and CKD is an important finding that strengthens the inferences that can be drawn from this work.

In conclusion, in this *post hoc* analysis of the CREDENCE trial, we observed that 1-year treatment with canagliflozin increased TIBC and reduced ferritin, suggesting the mobilization of stored iron for erythropoiesis. The beneficial effects of canagliflozin on anemia and clinical outcomes were consistent regardless of iron deficiency at baseline. These results support the use of SGLT2 inhibitors in patients with type 2 diabetes and CKD, regardless of iron deficiency.

## Supplementary Material

gfae198_Supplemental_File

## Data Availability

Data from this study will be made available in the public domain via the Yale University Open Data Access Project (http://yoda.yale.edu/).
