# Supplementary material for: Canagliflozin and iron metabolism in the CREDENCE trial
Source: Nephrol Dial Transplant. 2024 Sep 20;40(4):696–706. doi: 10.1093/ndt/gfae198 (PMC11960735; doi:10.1093/ndt/gfae198)
Supplement: gfae198_Supplemental_File [file gfae198_supplemental_file.docx]

**Supplementary Table S1.** Percent changes in iron markers in the canagliflozin and placebo group from baseline to week 52 by sex, anemia, eGFR and UACR categories

| **Biomarker** | **Geometric mean at baseline (95% CI)** | | **Geometric mean at week 52 (95% CI)** | | **Change from baseline to week 52, % (95% CI)** | | **Effect, % (95% CI)*** | **p** | **p for int** |
| --- | --- | --- | --- | --- | --- | --- | --- | --- | --- |
|  | **Cana** | **Placebo** | **Cana** | **Placebo** | **Cana** | **Placebo** |  |  |  |
| **Iron (µmol/L)** |  |  |  |  |  |  |  |  |  |
| Overall (N=2013) | 13.1  (12.7, 13.4) | 13.1  (12.8, 13.4) | 12.6  (12.2, 12.9) | 12.7  (12.4, 13) | -3.8  (-6.1, -1.5) | -3.2  (-5.6, -0.8) | -0.6  (-4.0, 2.9) | 0.73 |  |
| Sex |  |  |  |  |  |  |  |  |  |
| Women (n=677) | 11.7  (11.3, 12.2) | 11.6  (11.1, 12.1) | 11.2  (10.7, 11.6) | 11.4  (10.9, 11.9) | -4.6  (-8.2, -0.8) | -2.1  (-6, 1.8) | -2.5  (-7.8, 3.1) | 0.37 | 0.41 |
| Men (n=1336) | 13.8  (13.4, 14.2) | 13.9  (13.5, 14.3) | 13.4  (12.9, 13.8) | 13.3  (12.9, 13.7) | -3.4  (-6.3, -0.4) | -3.9  (-6.8, -0.9) | 0.5  (-3.7, 5) | 0.82 |  |
| Anemia |  |  |  |  |  |  |  |  |  |
| No (n=1289) | 14.1  (13.7, 14.5) | 14.1  (13.7, 14.5) | 13.3  (12.9, 13.8) | 13.4  (13.0, 13.8) | -5.5  (-8.2, -2.7) | -5.1  (-8.0, -2.2) | -0.4  (-4.5, 3.9) | 0.85 | 0.65 |
| Yes (n=720) | 11.2  (10.7, 11.7) | 11.6  (11.2, 12.1) | 11.2  (10.7, 11.7) | 11.6  (11.1, 12.1) | -1.2  (-5.4, 3.1) | 0.8  (-3.3, 5.0) | -2.0  (-7.7, 4.1) | 0.51 |  |
| eGFR, mL/min/1.73m^2^ |  |  |  |  |  |  |  |  |  |
| ≤60 (n=1194) | 12.8  (12.4, 13.2) | 12.9  (12.5, 13.3) | 12.3  (11.9, 12.8) | 12.3  (11.9, 12.7) | -4.3  (-7.2, -1.2) | -4.5  (-7.6, -1.4) | 0.3  (-4.1, 4.9) | 0.90 | 0.55 |
| >60 (n=819) | 13.4  (12.8, 14.0) | 13.4  (12.9, 13.9) | 13.0  (12.4, 13.5) | 13.2  (12.7, 13.7) | -3.2  (-6.7, 0.5) | -1.3  (-5.0, 2.5) | -1.8  (-6.9, 3.5) | 0.50 |  |
| UACR, mg/g |  |  |  |  |  |  |  |  |  |
| ≤1000 (n=1109) | 13.3  (12.9, 13.8) | 13.3  (12.9, 13.8) | 12.7  (12.2, 13.2) | 12.9  (12.5, 13.3) | -5.0  (-8.0, -1.8) | -3.3  (-6.5, 0.0) | -1.7  (-6.2, 3.0) | 0.47 | 0.48 |
| >1000 (n=912) | 12.7  (12.2, 13.2) | 12.8  (12.4, 13.3) | 12.4  (12.0, 13.0) | 12.4  (11.9, 12.9) | -2.4  (-5.9, 1.1) | -3.2  (-6.6, 0.4) | 0.8  (-4.2, 6.0) | 0.77 |  |
| **TIBC (µmol/L)** |  |  |  |  |  |  |  |  |  |
| Overall (N=2004) | 62.5  (61.7, 63.3) | 62.2  (61.3, 63.1) | 62.5  (61.6, 63.4) | 61.1  (60.3, 61.9) | 0.2  (-1.0, 1.4) | -1.9  (-3.0, -0.7) | 2.1  (0.4, 3.8) | 0.014 |  |
| Sex |  |  |  |  |  |  |  |  |  |
| Women (n=672) | 63.5  (62.1, 64.9) | 63.0  (61.4, 64.6) | 63.0  (61.4, 64.5) | 61.5  (60.0, 63.0) | -0.7  (-2.6, 1.2) | -2.5  (-4.4, -0.6) | 1.8  (-0.9, 4.7) | 0.19 | 0.86 |
| Men (n=1332) | 61.9  (61.0, 62.9) | 61.8  (60.8, 62.8) | 62.3  (61.2, 63.4) | 60.9  (59.9, 61.9) | 0.7  (-0.8, 2.1) | -1.5  (-3.0, -0.1) | 2.2  (0.1, 4.3) | 0.04 |  |
| Anemia |  |  |  |  |  |  |  |  |  |
| No (n=1286) | 63.5  (62.6, 64.5) | 63.4  (62.3, 64.5) | 63.5  (62.5, 64.5) | 62.2  (61.1, 63.2) | 0.0  (-1.3, 1.3) | -2.0  (-3.3, -0.6) | 2.0  (0.1, 3.9) | 0.04 | 0.99 |
| Yes (n=714) | 60.4  (59.0, 61.8) | 60.4  (58.9, 61.8) | 60.6  (58.9, 62.5) | 59.4  (58, 60.8) | 0.4  (-1.9, 2.8) | -1.6  (-3.7, 0.6) | 2.0  (-1.2, 5.3) | 0.22 |  |
| eGFR, mL/min/1.73m^2^ |  |  |  |  |  |  |  |  |  |
| ≤60 (n=1188) | 61.5  (60.4, 62.5) | 61.3  (60.2, 62.4) | 60.9  (59.7, 62.1) | 59.8  (58.7, 60.9) | -0.9  (-2.5, 0.7) | -2.6  (-4.1, -1.0) | 1.7  (-0.6, 4.0) | 0.14 | 0.57 |
| >60 (n=816) | 63.9  (62.7, 65.2) | 63.5  (62.2, 64.9) | 65.0  (63.7, 66.4) | 63.1  (61.8, 64.3) | 1.8  (0.2, 3.5) | -0.9  (-2.5, 0.8) | 2.7  (0.3, 5.2) | 0.03 |  |
| UACR, mg/g |  |  |  |  |  |  |  |  |  |
| ≤1000 (n=1100) | 64.6  (63.5, 65.7) | 63.3  (62.1, 64.5) | 65.1  (64.0, 66.2) | 62.4  (61.3, 63.4) | 1.2  (-0.2, 2.6) | -1.9  (-3.3, -0.5) | 3.2  (1.2, 5.3) | 0.002 | 0.17 |
| >1000 (n=904) | 60.0  (58.8, 61.1) | 60.9  (59.7, 62.1) | 59.5  (58.2, 60.9) | 59.6  (58.3, 60.9) | -1.0  (-2.9, 0.9) | -1.8  (-3.7, 0.1) | 0.9  (-1.9, 3.6) | 0.54 |  |
| **TSAT (%)** |  |  |  |  |  |  |  |  |  |
| Overall (N=2003) | 20.9  (20.4, 21.5) | 21.1  (20.6, 21.6) | 20.1  (19.5, 20.7) | 20.7  (20.2, 21.3) | -4.1  (-6.3, -1.8) | -1.5  (-3.8, 0.9) | -2.7  (-5.9, 0.6) | 0.11 |  |
| Sex |  |  |  |  |  |  |  |  |  |
| Women (n=672) | 18.5  (17.7, 19.3) | 18.4  (17.6, 19.3) | 17.7  (16.9, 18.6) | 18.6  (17.7, 19.4) | -4.2  (-7.9, -0.3) | 0.6  (-3.4, 4.7) | -4.7  (-10.0, 0.9) | 0.10 | 0.38 |
| Men (n=1331) | 22.3  (21.6, 23) | 22.5  (21.9, 23.2) | 21.4  (20.7, 22.2) | 21.9  (21.3, 22.6) | -4.1  (-6.8, -1.2) | -2.5  (-5.3, 0.4) | -1.6  (-5.6, 2.5) | 0.44 |  |
| Anemia |  |  |  |  |  |  |  |  |  |
| No (n=1285) | 22.2  (21.5, 22.9) | 22.3  (21.7, 23.0) | 21.0  (20.2, 21.7) | 21.5  (20.9, 22.2) | -5.6  (-8.3, -2.8) | -3.3  (-6.2, -0.3) | -2.4  (-6.4, 1.8) | 0.26 | 0.71 |
| Yes (n=714) | 18.6  (17.8, 19.5) | 19.2  (18.4, 20.1) | 18.5  (17.6, 19.4) | 19.5  (18.7, 20.4) | -1.4  (-5.4, 2.7) | 2.2  (-1.7, 6.2) | -3.6  (-8.8, 2.0) | 0.20 |  |
| eGFR, mL/min/1.73m^2^ |  |  |  |  |  |  |  |  |  |
| ≤60 (n=1187) | 20.9  (20.2, 21.6) | 21.1  (20.4, 21.8) | 20.2  (19.5, 21.0) | 20.6  (19.9, 21.3) | -3.4  (-6.3, -0.4) | -2.1  (-5.1, 0.9) | -1.3  (-5.4, 3.1) | 0.56 | 0.31 |
| >60 (n=816) | 21.0  (20.1, 21.9) | 21.1  (20.3, 21.9) | 19.9  (19.0, 20.9) | 20.9  (20.1, 21.8) | -5.2  (-8.7, -1.5) | -0.5  (-4.2, 3.4) | -4.7  (-9.7, 0.5) | 0.08 |  |
| UACR mg/g |  |  |  |  |  |  |  |  |  |
| ≤1000 (n=1100) | 20.7  (20.0, 21.4) | 21.1  (20.4, 21.8) | 19.4  (18.7, 20.3) | 20.7  (20, 21.4) | -6.4  (-9.3, -3.3) | -1.4  (-4.6, 1.9) | -5.0  (-9.3, -0.5) | 0.03 | 0.11 |
| >1000 (n=903) | 21.2  (20.4, 22.1) | 21.1  (20.3, 21.9) | 20.9  (20.1, 21.8) | 20.8  (20.0, 21.6) | -1.2  (-4.5, 2.3) | -1.6  (-4.9, 1.9) | 0.4  (-4.4, 5.4) | 0.88 |  |
| **Ferritin (µg/L)** |  |  |  |  |  |  |  |  |  |
| Overall (N=1834) | 130.5  (122.7, 138.7) | 129.4  (121.5, 137.7) | 108.2  (101.7, 115.1) | 121.4  (114.2, 129.0) | -17.0  (-19.8, -14.2) | -6.2  (-9.4, -3.0) | -11.5  (-15.7, -7.1) | <0.001 |  |
| Sex |  |  |  |  |  |  |  |  |  |
| Women (n=609) | 104.9  (94.5, 116.4) | 100.9  (90.2, 113) | 90.9  (81.7, 101.1) | 93.8  (84.1, 104.5) | -13.0  (-18.1, -7.6) | -7.4  (-13.1, -1.4) | -6.0  (-13.9, 2.6) | 0.16 | 0.09 |
| Men (n=1225) | 146.1  (135.6, 157.4) | 145.6  (135.3, 156.7) | 118.4  (109.9, 127.6) | 137.3  (127.8, 147.5) | -18.9  (-22.2, -15.6) | -5.7  (-9.5, -1.8) | -14,0  (-18.8, -8.9) | <0.001 |  |
| Anemia |  |  |  |  |  |  |  |  |  |
| No (n=1174) | 141.2  (131.5, 151.6) | 137.3  (127.2, 148.3) | 112.9  (105.0, 121.4) | 127.1  (117.8, 137.1) | -19.9  (-23.0, -16.6) | -7.7  (-11.5, -3.7) | -13.2  (-18.1, -8.0) | <0.001 | 0.26 |
| Yes (n=656) | 110.8  (98.7, 124.3) | 117.7  (105.7, 131.0) | 98.9  (88.2, 110.9) | 113.0  (102.0, 125.2) | -11.3  (-16.6, -5.6) | -3.4  (-8.9, 2.4) | -8.1  (-15.7, 0.1) | 0.052 |  |
| eGFR, mL/min/1.73m^2^ |  |  |  |  |  |  |  |  |  |
| ≤60 (n=1079) | 129.7  (119.9, 140.2) | 126.9  (117.4, 137.2) | 111.8  (103.5, 120.8) | 121.0  (112.2, 130.6) | -13.6  (-17.3, -9.7) | -4.8  (-9.0, -0.5) | -9.2  (-14.7, -3.3) | 0.003 | 0.21 |
| >60 (n=755) | 131.6  (119.2, 145.4) | 133.0  (119.9, 147.5) | 103.2  (93.2, 114.2) | 122.0  (110.2, 134.9) | -21.7  (-25.7, -17.5) | -8.2  (-13.0, -3.2) | -14.7  (-20.8, -8.1) | <0.001 |  |
| UACR, mg/g |  |  |  |  |  |  |  |  |  |
| ≤1000 (n=1013) | 126.7  (116.4, 137.9) | 127.3  (117.0, 138.5) | 102.1  (93.6, 111.4) | 117.8  (108.7, 127.6) | -19.4  (-23.0, -15.7) | -7.4  (-11.5, -3.1) | -13.0  (-18.4, -7.3) | <0.001 | 0.45 |
| >1000 (n=821) | 135.2  (123.7, 147.8) | 132.1  (120.2, 145.1) | 116.0  (106.4, 126.6) | 126.1  (114.8, 138.5) | -14.0  (-18.3, -9.5) | -4.7  (-9.6, 0.4) | -9.7  (-16.1, -2.8) | 0.006 |  |

* Placebo-corrected effects of canagliflozin on iron biomarkers were estimated with analysis of covariance adjusted for log-transformed value at baseline.

Anemia was defined as hemoglobin <130g/L in men or <120g/L in women.

Cana denotes canagliflozin; eGFR, estimated glomerular filtration ratio; TIBC, total iron binding capacity; TSAT, transferrin saturation; UACR, urine albumin-creatinine ratio.

**Supplementary Table S2.** Correlation coefficients between changes in hemoglobin/hematocrit and those in iron markers from baseline to week 52 by treatment group

| **Biomarker change** | **Placebo** |  | **Canagliflozin** |  |
| --- | --- | --- | --- | --- |
|  | **Correlation coefficient** | **p value** | **Correlation coefficient** | **p value** |
| **Hemoglobin change** |  |  |  |  |
| Iron change | 0.25 | <0.001 | 0.17 | <0.001 |
| TIBC change | 0.04 | 0.195 | 0.21 | <0.001 |
| TSAT change | 0.24 | <0.001 | 0.08 | 0.009 |
| Ferritin change | 0.07 | 0.031 | -0.07 | 0.049 |
| **Hematocrit change** |  |  |  |  |
| Iron change | 0.18 | <0.001 | 0.12 | <0.001 |
| TIBC change | 0.09 | 0.003 | 0.21 | <0.001 |
| TSAT change | 0.14 | <0.001 | 0.03 | 0.318 |
| Ferritin change | -0.02 | 0.552 | -0.12 | <0.001 |

Correlation coefficients were shown as Pearson`s R.

TIBC denotes total iron binding capacity; TSAT, transferrin saturation.

**Supplementary Table S3.** Effects of canagliflozin on hemoglobin and hematocrit by the presence of iron deficiency at baseline in patients with eGFR ≤60 mL/min/1.73m^2^ at screening.

|  | Mean at baseline (SD) | | Mean difference during follow-up (95%CI) * | *p* for interaction |
| --- | --- | --- | --- | --- |
|  | Canagliflozin | Placebo |  |  |
| Hemoglobin concentration, g/L | | | | |
| Whole subgroup (N=1303) | 130.5 (17.3) | 129.5 (17.0) | 7.2 (6.0, 8.4) |  |
| Not iron deficient (n=810) | 133.4 (17.2) | 132.3 (16.9) | 7.1 (5.6, 8.5) | 0.50 |
| Iron deficient (n=493) | 125.9 (16.6) | 124.6 (15.9) | 7.5 (5.5, 9.5) |  |
| Hematocrit concentration, % | | | | |
| Whole subgroup (N=1292) | 39.9 (5.4) | 39.6 (5.1) | 2.4 (2.1, 2.8) |  |
| Not iron deficient (n=804) | 40.6 (5.4) | 40.3 (5.1) | 2.3 (1.9, 2.8) | 0.63 |
| Iron deficient (n=488) | 38.8 (5.2) | 38.4 (5.0) | 2.7 (2.1, 3.3) |  |

*Canagliflozin group minus placebo group

Linear mixed-effects models with a restricted maximum likelihood estimator calculated the least-squares mean changes from baseline in hemoglobin and hematocrit. The model included fixed, categorical effects of therapy, trial visit, eGFR at screening, and treatment-by-visit interaction fixed along with fixed, continuous baseline value variables and baseline value by visit interaction. Effect modification by iron deficiency at baseline was tested by adding the main effect for iron deficiency subgroup (yes or no) and all two-way and three-way interaction terms between treatment, iron deficiency and trial visit to the relevant models. An unstructured covariance structure was used to model the within-patient errors.

**Supplementary Table S4.** Effect of canagliflozin on time to correction and onset of anemia by iron deficiency at baseline

|  | **Events, n (%)** | |  | **Events /1000PY** | | **HR (95%CI)** | ***p* value** | ***p* for interaction** |
| --- | --- | --- | --- | --- | --- | --- | --- | --- |
|  | **Canagliflozin** | **Placebo** |  | **Canagliflozin** | **Placebo** |  |  |  |
| **Anemia correction** |  |  |  |  |  |  |  |  |
| Overall population | 193/370 (52.2) | 104/401 (25.9) |  | 316.6 | 132.1 | 2.55 (2.01, 3.24) | <0.001 |  |
| Iron deficient |  |  |  |  |  |  |  |  |
| No | 83/175 (47.4) | 51/213 (23.9) |  | 276.9 | 124.2 | 2.43 (1.71, 3.45) | <0.001 | 0.78 |
| Yes | 110/195 (56.4) | 53/188 (28.2) |  | 355.1 | 140.7 | 2.63 (1.89, 3.66) | <0.001 |  |
| **Anemia onset** |  |  |  |  |  |  |  |  |
| Overall population | 161/739 (21.8) | 245/677 (36.2) |  | 97.7 | 178.4 | 0.51 (0.41, 0.62) | <0.001 |  |
| Iron deficient |  |  |  |  |  |  |  |  |
| No | 111/504 (22.0) | 158/458 (34.5) |  | 97.1 | 166.6 | 0.54 (0.42, 0.69) | <0.001 | 0.31 |
| Yes | 50/235 (21.3) | 87/219 (39.7) |  | 99.1 | 204.8 | 0.44 (0.31, 0.63) | <0.001 |  |

Anemia was defined as hemoglobin <130g/L in men or <120g/L in women. Anemia correction defined as a single measurement above the threshold in patients with anemia at baseline. Conversely, anemia onset was defined as a single measurement below the threshold in patients without anemia at baseline. Cox proportional hazard regression model was stratified with prespecified eGFR strata at screening.

CI denotes confidential interval; eGFR estimated glomerular filtration rate; HR, hazard ratio; PY, person year.

**Supplementary Figure S1.** CONSORT diagram describing the number of patients in each analysis
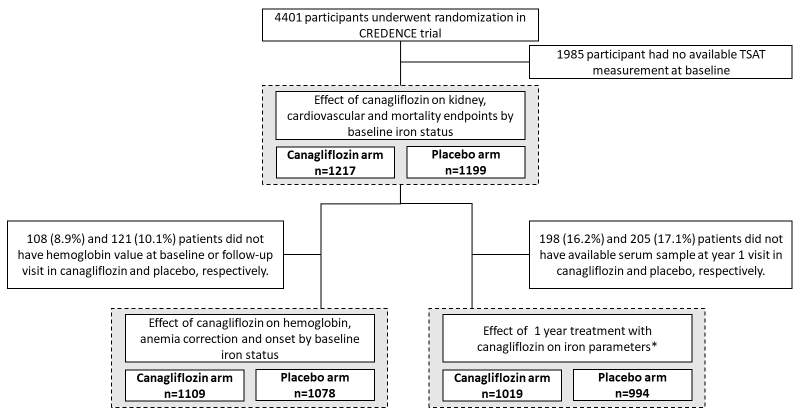


* 2013 participants have iron concentration at both baseline and year 1. Of those, 9, 10, and 179 had missing total iron binding capacity, TSAT, and ferritin measurements at year 1, respectively.

TSAT denotes transferrin saturation.

**S****upplementary Figure S2.** Proportion of patients with iron deficiency at baseline and week 52 by treatment group

ID was defined as TSAT<20%. Within iron deficiency, functional and absolute iron deficiency was categorized using cut-off of ferritin 100 µg/L. Numbers above each bar show percentage of ID patients. Proportion of ID patients between treatment groups at week 52 was compared using chi-square test.

ID denotes iron deficient.
